# Intact Glucocorticoid Receptor Dimerization Is Deleterious in Trauma-Induced Impaired Fracture Healing

**DOI:** 10.3389/fimmu.2020.628287

**Published:** 2021-02-17

**Authors:** Yasmine Hachemi, Anna E. Rapp, Sooyeon Lee, Ann-Kristin Dorn, Benjamin T. Krüger, Kathrin Kaiser, Anita Ignatius, Jan Tuckermann

**Affiliations:** ^1^ Institute of Comparative Molecular Endocrinology, Ulm University, Ulm, Germany; ^2^ Institute of Orthopedic Research and Biomechanics, Ulm University Medical Center, Ulm, Germany

**Keywords:** inflammation, glucocorticoid receptor, fracture, thoracic trauma, bone repair

## Abstract

Following severe trauma, fracture healing is impaired because of overwhelming systemic and local inflammation. Glucocorticoids (GCs), acting *via* the glucocorticoid receptor (GR), influence fracture healing by modulating the trauma-induced immune response. GR dimerization-dependent gene regulation is essential for the anti-inflammatory effects of GCs. Therefore, we investigated in a murine trauma model of combined femur fracture and thoracic trauma, whether effective GR dimerization influences the pathomechanisms of trauma-induced compromised fracture healing. To this end, we used mice with decreased GR dimerization ability (GR^dim^). The healing process was analyzed by cytokine/chemokine multiplex analysis, flow cytometry, gene-expression analysis, histomorphometry, micro-computed tomography, and biomechanical testing. GR^dim^ mice did not display a systemic or local hyper-inflammation upon combined fracture and thorax trauma. Strikingly, we discovered that GR^dim^ mice were protected from fracture healing impairment induced by the additional thorax trauma. Collectively and in contrast to previous studies describing the beneficial effects of intact GR dimerization in inflammatory models, we report here an adverse role of intact GR dimerization in trauma-induced compromised fracture healing.

## Introduction

Fracture healing is largely impaired in patients suffering from multiple injuries (1, 2). For example, multi-injured patients with tibial fractures exhibit a higher risk for non-union (2). Thoracic trauma particularly represents a critical injury in multi-injured patients and it frequently occurs in association with fractures. Indeed, 50% of patients with a blunt thoracic trauma suffer additional fractures of the extremities (3) and the mortality is markedly elevated in polytraumatized patients with severe thoracic trauma compared to patients with similar severity of injury without thoracic trauma (4). Thoracic trauma is also a strong inducer of the posttraumatic systemic inflammation with a rapid release of pro-inflammatory cytokines, including interleukin (IL)-6 (5, 6). Thoracic trauma can lead to acute lung injury, a strong inflammatory response in the lungs, which affects a subfraction of patients, leading to a systemic trigger that impacts on the whole organism (7). Several experimental models combining femur fracture and thorax trauma described a systemic inflammation and disturbed fracture healing (6, 8), indicating the interference of the posttraumatic inflammation in the fracture healing process (9). Endogenous glucocorticoids (GCs) are stress hormones that play a crucial role in controlling inflammation and maintaining bone mass (10, 11). We previously demonstrated that endogenous GCs signaling through the glucocorticoid receptor (GR) is critical for efficient fracture healing (12). However, less is known about the function of GCs during bone regeneration in the context of multiple injuries.

Endogenous GCs are released from the adrenal cortex in a circadian and stress-associated manner under the control of the hypothalamic-pituitary-adrenal axis (13). Their effects are mediated by the nuclear receptors for glucocorticoid and mineralocorticoid receptors. Upon binding of GCs, GR translocates into nucleus and acts as a homodimer or a monomer to regulate gene expression (11). The GR dimer can bind to GC-response elements in DNA and induce gene expression by transactivation (11). The GR monomer can bind directly to DNA or indirectly by tethering with transcriptions factors, including nuclear factor kappa-light-chain-enhancer of activated B cells (NF-κB) and activator protein 1 (AP-1), leading to transrepression of genes (14, 15). It was previously considered that transrepression was the major mechanism of GC’s anti-inflammatory effects (16). Therefore, a mouse model with impaired dimerization of the GR (GR^dim^) was generated (17) by introducing a point mutation into the second zinc finger, which encompasses one of the dimerization interfaces. In these GR^dim^ mice, GCs failed to suppress inflammation during allergy (18), acute lung injury (19), and systemic inflammation (20, 21), revealing a critical role for GR dimerization in controlling inflammation by transactivation of gene expression. Moreover, GR^dim^ mice still develop osteoporosis when treated with pharmacological doses of GCs indicating that these effects are mediated through the GR monomer and its transrepression activity (22).

Endogenous GCs signaling is essential for maintaining bone homeostasis, because patients with adrenal insufficiency exhibit an increased fracture risk (23) and mice with selective GR deletion in the osteoblast lineage have a reduced trabecular bone mass (22). Endogenous GCs signaling plays a crucial role in osteoblastogenesis through the activation of Wnt signaling (24). Few studies have addressed the role of endogenous GCs during fracture healing. We and others could demonstrate a critical role for the GR during endochondral ossification (12, 25). Whether intact GR dimerization is important for proper fracture healing remains unknown. Thoracic trauma induces a systemic posttraumatic inflammation that potentially interferes with fracture healing (5, 6). We hypothesize that intact GR dimerization is important for proper fracture healing and for the overall control of the systemic posttraumatic inflammation caused by additional thoracic trauma. To test this hypothesis, we used mice with impaired GR dimerization capacity (GR^dim^) and subjected them to isolated femur fracture as a model of uncomplicated healing or to combined fracture and thoracic trauma as a model of inflammation-induced disturbed healing.

## Material and Methods

### Study Design

We performed femur osteotomy with or without thoracic trauma on 14-week old male GR^dim^ mice and littermate wildtype controls (**Supplementary Figure 1A**). Mice were euthanized at different time points during fracture healing (**Supplementary Figure 1B**). At 6 h, 24 h, and 10 days post operation, we analyzed lung tissues by HE staining and qRT-PCR to evaluate the effects of the trauma. At 24 h post operation, blood plasma, bronco-alveolar lavage (BAL) and hematoma samples were processed for cytokine and chemokine measurement by multiplex assay. Immune cell profile from blood and hematoma samples was also analyzed by FACS. Callus of the fractured femur was evaluated at day 10 and day 23 by histology, histomorphometry, microCT and biomechanical analysis.

Sample size of 8 per group was determined by G*Power software (Version 3.1.9.6; Heinrich Heine University, Düsseldorf, Germany) with 80% power and a significance level of 5% based on previous data (12) taking flexural rigidity as the primary readout. We used T-test to compare between two independent groups.

Exclusion criteria included: death following thorax trauma, over-range osteotomy gap size, skins wounds resulting from bites and shattered bone during the osteotomy procedure. The animals were randomly assigned to each group and assessment of the outcome was performed blindly.

### Animal Model and Husbandry

The animal experiments were performed in compliance with the international regulations for the care and use of laboratory animals (Directive 2010/63/EU) and with the approval of the local ethical committee (Regierungspräsidium Tübingen, Germany Reg. No. 1225). GR^dim^ mice (Nr3c1^tm3Gsc^) were maintained in the BALB/c background.

Genotypes were determined by polymerase chain reaction (PCR) using DNA from tail biopsies as described previously (22). The mice were housed in groups of up to five animals with a 14-h light, 10-h dark cycle at 23°C and 55 ± 10% humidity. Standard rodent chow and water were available *ad libitum*. Mice were housed in specific pathogen free (SPF) conditions during breeding and transferred to experimental area in individually ventilated caging (IVC).

When aged 14 weeks, male mice received an unilateral femur osteotomy as described previously (26). In brief, under general anesthesia (2 vol% isoflurane, Forene^®^, Abbott, Wiesbaden Germany), the right femur was exposed and the mid shaft was osteotomized using a 0.4-mm Gigli saw (RISystem, Davos, Switzerland). The osteotomy was stabilized using an external fixator (axial stiffness 3.2 N/mm, RISystem, Davos, Switzerland) that was fitted to the bone with four mini-Schanz screws. The osteotomy was combined with an additional thoracic trauma as described previously (27) to induce systemic inflammation. The thoracic trauma directly followed the femur osteotomy, while the mice were still under general anesthesia. In brief, a blast wave generator placed 2 cm from the mid-thorax released a blast wave at 13 bar pressure, inducing a standardized bilateral, isolated lung contusion. An analgesic was administered *via* the drinking water from 1 day before to 3 days after surgery (tramadol-hydrochloride, 25 mg/l; Gruenenthal, Aachen, Germany). Prior to surgery, mice received a single dose of antibiotics (clindamycin-2-dihydrogenphosphate, 45 mg/kg; Ratiopharm, Ulm, Germany). Mice were euthanized by intracardial blood withdrawal under deep isoflurane anesthesia after 6 or 24 h, and after 10 or 23 days.

### Cytokine and Chemokine Analysis

Blood serum, broncho-alveolar lavage (BAL) fluid, and fracture hematomas harvested 24 h after osteotomy were used for cytokine analysis. Blood was collected in microvettes (Sarstedt AG&Co. Nümbrecht, Germany) and centrifuged at 4,000 *g* for 10 min at 4°C. The obtained plasma was stored at −80°C until use. BAL fluid was obtained, after flushing the lungs with 0.5 ml of ice-cold phosphate-buffered saline solution. In total, 5 µl protease inhibitor mixture were added to the obtained BAL fluid and the samples were stored on ice. The samples were centrifuged at 300 *g* for 15 min and the supernatants were harvested and kept at −80°C until use. The fracture hematoma was directly transferred into lysis buffer (10 mM tris pH 7.5, 10 mM NaCl, 0.5 mM Triton X-100, 0.2 mM phenylmethylsulfonyl fluoride, all from Sigma-Aldrich, Steinheim, Germany) with protease inhibitor cocktail (Halt™ Protease and Phosphatase Inhibitor Cocktail, Fisher Scientific GmbH, Schwerte, Germany) and minced. Following 30 min incubation on ice, the samples were centrifuged at 18,000 *g* for 30 min at 4°C. The protein concentration was determined using the Pierce™ BCA Protein Assay Kit (ThermoFisher Scientific GmbH) according to the manufacturer’s instructions. The lysates were stored at −80°C until use. Using a mouse multiplex cytokine and chemokine assay (ProcartaPlex™, eBioscience, San Diego, CA, USA), the levels of the pro-inflammatory mediators IL-6, monocyte chemotactic protein (MCP)-1, macrophage inflammatory protein (MIP)-1α, and keratinocyte chemoattractant (CXCL-1) were determined on a Bio-Plex 200 (Bio-Rad, Hercules, CA, USA). Using standard curves, the cytokine levels were calculated automatically (Bio-Plex Manager™ software).

### Flow Cytometry

At 24 h after osteotomy, blood and the fracture hematoma were analyzed for immune cells by flow cytometry. To remove erythrocytes from blood, lysis was performed twice for 5 min on 37°C using lysis buffer (150 mM NH_4_Cl, 10 mM KHCO_3_, 0.125 mM ethylenediamintetraacetic acid (EDTA), all from Sigma-Aldrich). The hematoma between the bone ends was excised and pressed through a cell strainer to obtain a single-cell suspension. Cells of the innate and adaptive immune systems were stained using the following antibodies or respective isotypes: CD11b Alexa Fluor 700 (clone M1/70, eBioscience, 1:400 dilution), Ly-6G V450 (clone 1A8, BD Pharmingen, 1:400 dilution), F4/80 FITC (clone BM8, eBioscience, 1:50 dilution), CD3ϵ PE-Cyanine 7 (clone 145-2C11, eBioscience, 1:100 dilution), CD4 APC-eFluor 780 (clone GK1.5, eBioscience, 1:200 dilution), CD8a APC (clone 53-6.7, eBioscience, 1:800 dilution), and CD19 PE (clone 1D3, eBioscience, 1:400 dilution). 7-AAD (Sigma) was used to distinguish between living and dead cells. A minimum of 10,000 events was measured on an LSRII flow cytometer (BD Biosciences, San Jose, CA, USA) and analyzed using FlowJo software (FlowJo, Ashland, OR, USA). Detailed gating strategy is provided in **Supplementary Figure 1C**.

### Histology and Histomorphometry

Harvested lungs at 6 h and 10 days were fixed in 4% formaldehyde, embedded in paraffin, and stained with hematoxylin and eosin (H&E, Mayer’s hemalum solution Merck KGaA^®^, Darmstadt, Germany and Eosin Y, Applichem, Darmstadt, Germany) for morphological investigations.

Femurs harvested at different time points were fixed for 48 h, decalcified in 20% EDTA for 10–12 days, dehydrated, and embedded in paraffin. Sections of 6–8 µm thickness were stained using safranin-O/fast green. Evaluation of the callus composition was performed by light microscopy (Leica DMI6000 B; Software MetaMorph^®^, Leica Microsystems, Mannheim, Germany) under 50-fold magnification. At all the time points, the entire callus was analyzed for the amount of bone, cartilage, and fibrous tissue. To determine the number of osteoblasts, sections were stained with toluidine blue. For visualization of osteoclasts, tartrate-resistant acid phosphatase (TRAP) activity was determined by naphthol AS-MX phosphate and Fast Red TR-Salt (both Sigma-Aldrich) in 0.2 M acetate buffer pH 5.0. Osteoblast and osteoclast numbers and surface per bone surface were determined using the OsteoMeasure histomorphometry system (OsteoMetrics, Decatur, USA). Analysis was performed according to the recommendations of the American Society for Bone and Mineral Research (28).

### RNA Isolation and Gene Expression Analysis

RNA from lung tissue was isolated at 6 h and 10 days after isolated fracture or combined fracture and thoracic trauma using Trizol reagent (Invitrogen, Ambion, USA). Lungs were homogenized in Trizol for 3 min at 1,500 rotations/min using Precellys 24 homogenizer (Bertin Technologies, Montigny-le-Bretonneux, France), incubated at room temperature for 5 min, and 200 μl chloroform was added to the Trizol followed by centrifugation at maximum speed at 4°C. The aqueous phase was mixed with 0.7 volumes of isopropanol and centrifuging at maximum speed to pellet down RNA. The RNA pellet was washed with 500 μl 70% ethanol and dried at room temperature for 5–10min before suspending with RNase free water. RNA quality and amount were determined using the Nanodrop 2000 system (Thermo Scientific, Waltham, USA). cDNA was synthesized from 1–2 µg RNA using RevertAid H Minus reverse transcriptase (M-MulV, Fermentas, MA, USA) with 5× reverse transcriptase reaction buffer, random primers (200 ng/μl, Invitrogen, Carlsbad, CA, USA), and 10 mM deoxyribonucleotide triphosphate (dNTPs) following the manufacturer’s instructions. Quantitative real-time PCR (qRT-PCR) was performed in 10 μl reaction volume using SYBR Green I dye (Invitrogen) on an ABI ViiA-7 system (Applied Biosystems, Foster City, CA, USA). The mRNA abundance of *Nos2, Cd86, Il4, Chil3*, and *Il13* was calculated relative to the expression of the reference gene *Gapdh* and the data analyzed using a model based on correction for exact PCR efficiencies. The primers used are listed in **Supplementary Table 1**.

### Micro-Computed Tomography (µCT)

Osteotomized and intact femurs harvested after 23 days were scanned using a µCT device (Skyscan 1172, Bruker, Kontich, Belgium) at a resolution of 8 µm at a peak voltage of 50 kV and 200 µA. Within each scan, two phantoms with defined hydroxyapatite (HA) content (250 and 750 mg/cm^3^) were scanned to determine the bone mineral density (BMD). In intact femurs, a volume of interest of 1 mm length below the trochanter major was analyzed for cortical thickness (Ct.Th), mineralization (BMD), and 2^nd^ moment of inertia (Ix). Trabecular bone was analyzed in the distal femur 0.2 mm above the growth plate. In fractured femurs, the former osteotomy gap was analyzed for trabecular parameters. To distinguish between mineralized and non-mineralized tissue, a fixed global threshold set at 643 mg HA/cm^3^ was applied (29, 30). Basal bone phenotype was evaluated in intact femur and L5 vertebrae by measuring trabecular and/or cortical parameters at the age of 14 weeks. Analogous to the standard clinical assessment of X-rays to determine the healing outcome, the number of bridged cortices was determined in two perpendicular axes from µCT scans. A fracture was considered to be “healed” when the osteotomy gap was bridged at ≥3 sites (31).

### Biomechanical Testing

Intact and osteotomized femurs harvested after 23 days were analyzed for flexural rigidity by nondestructive three-point bending (26). In brief, the proximal end of the femur was fixed to an aluminum cup, which in turn was fixed to a hinge joint of the three-point bending setup in the material testing machine (Z10, Zwick Roell, Ulm, Germany). The femur condyles rested unfixed on a bending support. The bending load was applied to the mid-shaft of intact bones or the middle of the callus up to a maximum load of 4 N. Flexural rigidity (EI) was calculated from the slope (k) of the linear region of the force-displacement curve. The distance of the load vector and the proximal (a) and distal (b) bending supports was considered in the calculation when the force was not applied exactly in the middle between the supports (l/2). For the calculation, the formula EI=k(a^2^b^2^)/3l was used.

### Statistics

Statistical analyses were performed using GraphPad Prism7 (GraphPad Software Inc., La Jolla, CA, USA). Non-parametric Wilcoxon-Mann-Whitney or Kruskal-Wallis with Dunn’s multiple comparison test were used. *p<0.05, **p<0.01, ***p<0.005, ****p<0.001, was considered statistically significant.

## Results

### Trabecular Bone Mass Is Moderately Affected in Mice With Impaired GR Dimerization

**Figure 1:**
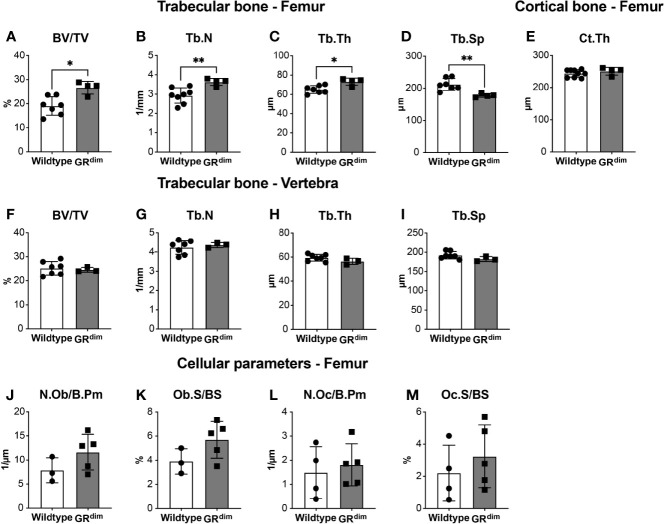
Trabecular but not cortical bone mass is moderately affected in mice with impaired glucocorticoid receptor (GR) dimerization. Bone phenotype of 14-week-old wild type and GR^dim^ mice was analyzed. Bone per tissue volume [BV/TV, **(A)**], trabecular number [Tb.N, **(B)**], trabecular thickness [Tb.Th, **(C)**], and trabecular separation [Tb.Sp, **(D)**] in the distal femur and L5 vertebrae **(F–I)** and cortical thickness of femur diaphysis [Ct.Th, **(E)**] were analyzed by micro-computed tomography. Osteoblast number per bone perimeter [Ob.N/B.Pm, **(J)**], osteoblast surface per bone surface [Ob.S/BS, **(K)**], osteoclast number per bone perimeter [Oc.N/B.Pm, **(L)**], and osteoclast surface per bone surface [Oc.S/BS, **(M)**] were analyzed by histomorphometry in the distal femur. Data are presented as the mean ± standard deviation. n=3–7 per group. *p<0.05, **p<0.01 using Mann-Whitney-Wilcoxon test.

Before subjecting GR^dim^ mice to fracture or combined fracture and thoracic trauma, we first analyzed the basal bone phenotype in these mice with a congenital deficiency of the GR dimer when aged 14 weeks. MicroCT analysis of the distal femur revealed a significantly higher bone volume per tissue volume (BV/TV) ratio in GR^dim^ mice compared to wild types (**Figure 1A**). Trabecular number (Tb.N) and thickness (Tb.Th) were significantly increased and accordingly trabecular spacing (Tb.Sp) was reduced in the femurs of GR^dim^ mice (**Figures 1B–D**). Cortical thickness of the femur diaphysis did not differ between mutant and wild type mice (**Figure 1E**). By contrast, in the L5 vertebrae there was no significant difference in the BV/TV, Tb.N, Tb.Th, or Tb.Sp between GR^dim^ and wild type mice (**Figures 1F–I**). Histomorphometry analysis of femur trabecular bone did not show any significant difference in terms of osteoblast or osteoclast parameters between GR^dim^ mice and wild type controls (**Figures 1J–M**). In summary, impairment of GR dimerization moderately increased trabecular bone mass in the femur but not in vertebrae, and this difference cannot be explained by a difference in osteoblast or osteoclast parameters.

### Impaired GR Dimerization Moderately Affects Inflammation in a Model of Combined Fracture and Thoracic Trauma

**Figure 2:**
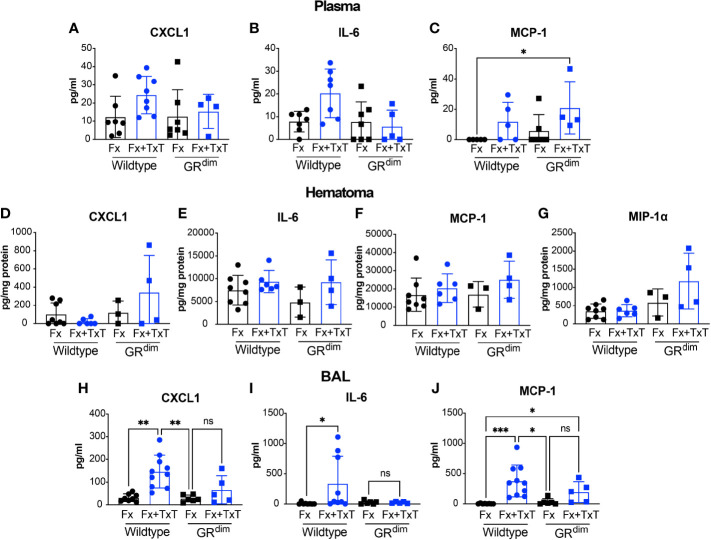
Impaired dimerization of the glucocorticoid receptor (GR) affects inflammation. Cytokine and chemokine levels in plasma, broncho-alveolar lavage (BAL), and callus homogenates were determined by cytokine and chemokine multiplex analysis 24 h after isolated fracture (Fx) or combined fracture and thorax trauma (Fx+TxT) in wild type (WT) and decreased GR dimerization ability (GR^dim^) mice. Levels of CXCL-1 **(A)**, IL-6 **(B)**, and MCP-1 **(C)** were measured in Plasma, Hematoma **(D–G)** and BAL **(H–J)**. In addition, MIP-1α was measured in the hematoma **(G)**. Data are presented as the mean ± standard deviation. n=4–7 per group Values are present in **Supplementary Data Sheet 1**. *p<0.05, **p<0.01, ns: non signficant, using ANOVA and Kruskal-Wallis tests.

Next, we applied a thoracic trauma concomitant with a femur fracture to analyze the consequences of a severe injury on fracture healing in GR^dim^ mice during different phases of the healing response. Twenty-four hours following isolated fracture (Fx) or combined fracture and thoracic trauma (Fx+TxT), we found that the chemokines CXCL-1, IL-6, and MCP-1 in plasma had increased by trend in wild type mice receiving additional thoracic trauma to the initial fracture compared to the wild type mice that had fracture alone (**Figures 2A–C**). At the fracture site (hematoma), there were no major alteration in levels of CXCL-1, IL-6, and MCP-1 (**Figures 2D–F**) except for MIP-1α that was higher by trend in GR^dim^ compared to wild type mice in the context of combined trauma (**Figure 2G**). The additional thoracic trauma significantly increased the concentration of CXCL-1, IL-6 and MCP-1 in the BAL fluid of wild type mice compared to the mice with fracture alone (**Figures 2H–J**). This effect was more subtle in GR^dim^ mice.

Flow cytometry analysis of immune cells in the blood and hematoma (**Table 1**) revealed no significant changes in polymorphonuclear leukocytes (PMNs) (CD11b^+^Ly6G^+^), monocytes/macrophages (CD11b^+^F4/80^+^), B-cells (CD19^+^), or T-cells (CD3^+^) between the different groups, indicating no substantial inflammation 24 h after fracture or combined trauma (**Table 1**).

**Table 1:** Frequency of immune cells in the blood and hematoma after isolated fracture or combined fracture and thorax trauma is not altered in mice with impaired glucocorticoid receptor (GR) dimerization.

Compartment	Cell populations	WT Fx n=3-5	WT Fx+TxT n=5	GR^dim^ Fx. n=2	GR^dim^ Fx+TxT n=3
Hematoma	PMNs (CD11b^+^Ly6G^+^)	8.8 ± 9.1	6.2 ± 4.1	9.7 ± 5.5	4.0 ± 1.8
	Monocytes/macrophages (CD11b^+^F4/80^+^)	3.8 ± 2.3	2.8 ± 3.3	2.6 ± 0.3	1.4 ± 0.3
	B-cells (CD19^+^)	3.2 ± 4.1	3.3 ± 3.1	6.9 ± 0.4	2.3 ± 1.5
	T-cells (CD3^+^)	2.9 ± 1.9	1.8 ± 7.5	2.8 ± 1.6	1.3 ± 0.5
	T-helper (CD3^+^CD4^+^)	0.3 ± 0.30	0.19 ± 0.29	0.07 ± 0.06	0.00092 ± 0.001
	Cytotoxic T-cells (CD3^+^CD8^+^)	0.022 ± 0.02	0.010 ± 0.012	0.012 ± 0.0015	0.0042 ± 0.0033
Blood	PMNs (CD11b^+^Ly6G^+^)	39.3 ± 29.8	53.56 ± 16.2	53.6 ± 22.9	59.8 ± 5.0
	Monocytes/macrophages (CD11b^+^F4/80^+^)	1.5 ± 0.7	2.0 ± 1.1	1.9 ± 1.2	1.8 ± 0.9
	B-cells (CD19^+^)	20.7 ± 12.4	10.2 ± 3.8	17. 9 ± 15.2	10.9 ± 4.8
	T-cells (CD3^+^)	18.0± 8.9	15.5 ± 6.4	11.2 ± 2.4	9.7 ± 1.6
	T-helper (CD3^+^CD4^+^)	13.4 ± 6.3	11.5 ± 4.83	8.7 ± 1.41	7.59 ± 1.6
	Cytotoxic T-cells (CD3^+^CD8^+^)	4.2 ± 2.5	3.6 ± 2.04	2.1 ± 0.78	1.57 ± 0.4

Data are presented as the mean ± standard deviation.

Immune cell populations circulating in the blood and the fracture hematoma were analyzed by flow cytometry 24 h after isolated fracture (Fx) or combined fracture and thorax trauma (Fx+Txt) in wild type (WT) and decreased GR dimerization ability (GR^dim^) mice.

Taken together, at the early time points investigated, the impaired GR dimerization had modest effects on the systemic inflammation that were most evident in the context of combined fracture and thoracic trauma.

### Additional Thoracic Trauma Differently Affects Lungs of the GR^dim^ Mice

**Figure 3:**
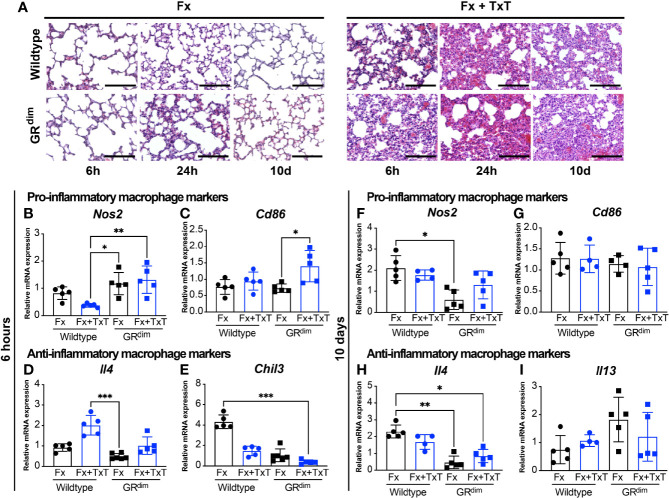
Additional thorax trauma decreases the expression of anti-inflammatory macrophage marker genes in the lungs of mice with impaired GR dimerization. H&E staining of lung sections illustrate the structural damage and the inflammation induced by the thoracic trauma 6 and 24 h and 10 days after isolated fracture (Fx) or combined fracture and thorax trauma (Fx+TxT) in wild type and GR^dim^ mice **(A)** scale bar in all micrographs: 100 μm. Gene expression of inducible nitric oxide synthase (*Nos2*) **(B, F)**, cluster of differentiation 86 (*Cd86*) **(C, G)**, interleukin-4 (*Il4*) **(D, H)**, chitinase-3-like protein 3 (*Chil3*) **(E)**, and *Il13* **(I)** were measured in the lungs 6 h and 10 days after Fx or FX+TxT by qRT-PCR. Data are presented as the mean ± standard deviation. n=4–5 per group, *p<0.05, **p<0.01, ***p<0.001, using ANOVA and Kruskal-Wallis tests.

Subsequently, we investigated the consequences of thoracic trauma on the lungs in wild type and GR^dim^ mice. We performed H&E staining to evaluate the lung structure at different time points: 6 h, 24 h, and 10 days (**Figure 3A**). As expected, histological analysis of the lungs from mice with isolated fracture displayed only minor structural signs of inflammation, including alveolar wall thickening, regardless of the genotype and time point, possibly resulting from the isoflurane overdose during sacrifice. In the context of the combined trauma, evident damage to the lung structure was observed in both GR^dim^ and wild type mice at early time points, with more exacerbated inflammatory cell infiltration at 24 h predominantly in GR^dim^ mice (**Figure 3A**). Blunt chest trauma created a non-homogenous injury pattern in the lungs, where some parts displayed massive damage with alveoli disruption and leukocyte infiltration, whereas other parts remained intact (**Supplementary Figure 2**).

To understand the role of macrophages during inflammation and repair, we evaluated the expression of particular marker genes of pro-inflammatory macrophages and anti-inflammatory macrophages in the lung tissue by qRT-PCR. Six hours post-injury, there was reduction, by trend, in *Nos2* gene expression for inducible nitric oxide synthase (iNOS), a marker for pro- inflammatory macrophages, in wild type controls while in GR^dim^ mice levels were high in the context of combined fracture and thoracic trauma (**Figure 3B**). *Cd86*, another pro-inflammatory marker, was also expressed significantly higher in the lung of GR^dim^ mice after combined trauma in comparison with GR^dim^ mice receiving only isolated fracture (**Figure 3C**). Regarding anti-inflammatory macrophage markers, *Il4* was up-regulated, by trend, upon additional thoracic trauma in wild type mice while this upregulation was more modest in GR^dim^ mice in which levels remained lower regardless of the injury type (**Figure 3D**). The anti-inflammatory marker *Chil3*, chitinase-like 3 (Ym1), was reduced by additional thoracic trauma in both wild type and GR^dim^ mice but levels of *Chil3* were lower in GR^dim^ compared to wild type mice, in both, isolated fracture and combined fracture and thoracic trauma (**Figure 3E**). At later healing stages, we did not detect changes in the expression of pro-inflammatory macrophage markers (*Nos2* or *Cd86*) 10 days post isolated fracture or combined fracture and thoracic trauma (**Figures 3F, G**). However, the anti-inflammatory macrophage marker *Il4* coding for the anti-inflammatory cytokine IL-4, was less expressed in the lung tissue of GR^dim^ mice both after isolated fracture and combined fracture and thoracic trauma (**Figure 3H**), similarly *Il13* as an anti-inflammatory molecule was not significantly altered (**Figure 3I**).

Overall, these results suggest that the thoracic trauma impaired lung tissue structure regardless of the mouse genotype. In addition, marker genes for pro-inflammatory macrophages appear to be expressed more in the early stage of inflammation whereas anti-inflammatory ones remained low throughout the inflammatory phase in the lungs of GR^dim^ mice.

### Impaired GR Dimerization Modulates Callus Formation

**Figure 4:**
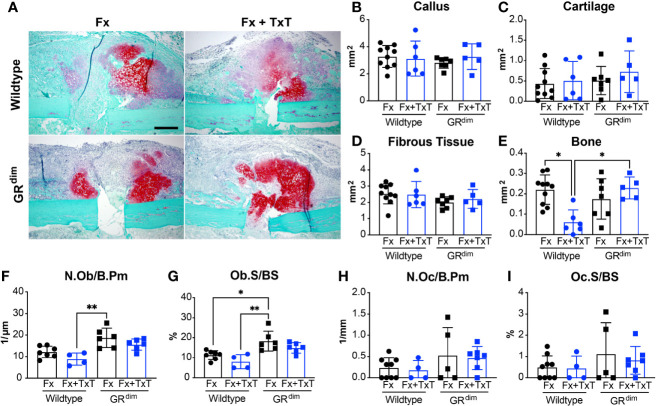
Callus maturation is not impaired while osteoblast number and osteoblast surface are higher in mice with impaired glucocorticoid receptor (GR) dimerization 10 days after combined fracture and thoracic trauma. Sections were stained using safranin-O/fast green to illustrate callus composition at day 10 after isolated fracture (Fx) or combined fracture and thorax trauma (Fx+TxT) **(A)**. In wild type and GR^dim^ mice, the callus area **(B)** and its composition: cartilage area **(C)**, fibrous tissue area **(D)**, and bone area **(E)** were analyzed on day 10 by histomorphometry. Osteoblast number per bone perimeter [Ob.N/B.Pm, **(F)**], osteoblast surface per bone surface [Ob.S/BS, **(G)**], osteoclast number per bone perimeter [Oc.N/B.Pm, **(H)**], and osteoclast surface per bone surface [Oc.S/BS, **(I)**] were assessed in the callus by histomorphometry. Scale bar in all micrographs: 200 μm. Data are presented as the mean ± standard deviation. n=4–10 per group. *p<0.05, **p<0.01 using ANOVA and Kruskal-Wallis tests.

To evaluate the intermediate healing response in the femur, we performed histomorphometry analysis on safranin-O/fast green-stained sections of the fracture callus at day 10 (**Figure 4A**). There were no significant differences in the callus size or cartilage or fibrous tissue content between the different groups (**Figures 4B–D**). However, the bone content was, as expected, significantly reduced in wild type mice in the combined trauma compared to the ones with isolated fracture (**Figure 4E**). Strikingly, the bone content in GR^dim^ mice was not reduced by the additional thoracic trauma and remained significantly higher compared to the wild type mice receiving combined trauma (**Figure 4E**). In addition, osteoblast number and surface were significantly higher in the fracture callus of GR^dim^ compared to wild type mice after both, isolated fracture and combined fracture and thoracic trauma (**Figures 4F, G**). By contrast, osteoclast parameters were not significantly different between the groups (**Figures 4H, I**).

Taken together, impaired GR dimerization had no effect on the endochondral ossification phase after isolated fracture. By contrast, the impaired GR dimerization protected against the disturbed callus maturation observed in the wild type mice upon additional thoracic trauma. The greater number of osteoblasts in the callus of GR^dim^ mice may at least in part explain their higher bone content.

### Mice With Impaired GR Dimerization Are Protected From the Detrimental Effects of Additional Thorax Trauma

**Figure 5:**
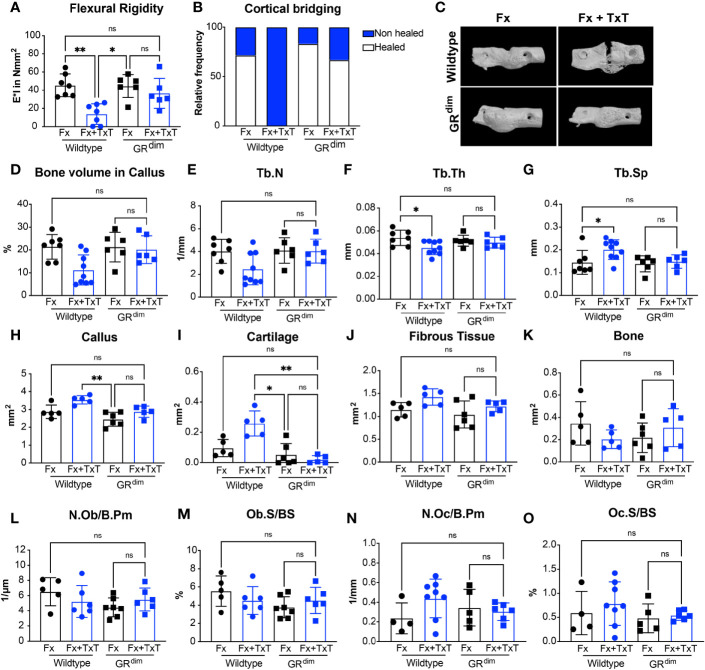
Mice with impaired glucocorticoid receptor (GR) dimerization are protected from the detrimental effect of additional thorax trauma. At day 23 after isolated fracture (Fx) or combined fracture and thorax trauma (Fx+TxT), flexural rigidity of the fracture calli was analyzed by a three-point bending test **(A)**. The healing frequency was assessed from micro-computed tomography (µCT) datasets **(B)** and three-dimensionally reconstructed fracture callus representations were generated **(C)**. Bone volume per tissue volume [BV/TV, **(D)**], trabecular number [Tb.N, **(E)**], trabecular thickness [Tb.Th, **(F)**], and trabecular spacing [Tb.Sp, **(G)**] were determined in the former osteotomy by µCT. Callus **(H)** and cartilage **(I)**, fibrous tissue **(J)**, and bone areas **(K)** were analyzed on day 23 by histomorphometry. Osteoblast number per bone perimeter [Ob.N/B.Pm, **(L)**], osteoblast surface per bone surface [Ob.S/BS, **(M)**], osteoclast number per bone perimeter [Oc.N/B.Pm, **(N)**], and osteoclast surface per bone surface [Oc.S/BS, **(O)**] were assessed in the callus by histomorphometry. Data are presented as the mean ± standard deviation. n=5–8 per group. *p<0.05, **p<0.01, ***p<0.001, ns: non significant using ANOVA and Kruskal-Wallis tests.

To evaluate the fracture healing outcome after isolated fracture and combined fracture and thoracic trauma in both genotypes, the bending stiffness of the fractured femurs was assessed at day 23. In the context of an isolated fracture, there was no difference between GR^dim^ and wild type mice (**Figure 5A**). However, after additional thoracic trauma, wild type mice displayed a significant reduction in the flexural rigidity, indicating impaired healing, which was not observed in GR^dim^ mice (**Figure 5A**). Accordingly, results of the cortical bridging analysis showed a higher proportion of successfully healed femurs in GR^dim^ compared to wild type mice in the context of combined trauma (**Figure 5B**), whereas in the context of an isolated fracture, there were no differences in comparison to wild type mice. These results were also reflected in the three-dimensional reconstructions of fracture calli (**Figure 5C**).

MicroCT analysis confirmed a higher bone content, by trend, in the fracture calli in GR^dim^ compared to wild type mice after combined trauma (**Figure 5D**). In agreement with this, the number of the trabeculae was higher and the trabecular spacing was reduced in GR^dim^ compared to wild type mice after combined trauma (**Figures 5E, G**). Additional thoracic trauma reduced trabecular thickness in wild type mice but had no significant effect on GR^dim^ mice (**Figure 5F**).

Histomorphometry of fracture calli at day 23 indicated that additional thoracic trauma tends to induce a larger callus area in wild type mice, whereas GR^dim^ mice exhibited a smaller callus size (**Figure 5H**). The differences in callus size could be due to the higher cartilage content, because there was significantly more cartilage in wild type mice after combined trauma, indicating delayed healing (**Figure 5I**). Additionally, no difference was detected between GR^dim^ and wild type mice in terms of callus size or cartilage content in the context of isolated fracture (**Figures 5H, I**). Fibrous tissue and bone tissue content did not exhibit major alterations in their composition between the different groups at this stage (**Figures 5J, K**). In parallel, osteoblast and osteoclast counts did not significantly differ between GR^dim^ and wild type mice 23 days post isolated fracture or combined trauma (**Figures 5L–O**).

In summary, these data indicate that GR^dim^ mice are protected from the adverse effect of additional thoracic trauma on bone fracture healing, implying an important role for the GR dimer in meditating these effects.

## Discussion

The present study investigated the role of endogenous GC signaling through the dimeric GR during both, isolated and compromised fracture healing induced by an additional thoracic trauma. Our results confirm previous findings on the detrimental effect of thoracic trauma on bone healing in wild type mice. In contrast to previous studies describing strong anti-inflammatory effects of intact GR dimerization in inflammatory models, we report here a negative effect of the GR dimer in trauma-induced compromised fracture healing.

GR^dim^ mice carry a point mutation in one of the dimerization interfaces of the GR that leads to a partial disruption of GR dimerization, which although incomplete (32), is still sufficient in the *in vivo* context to display impaired binding to palindromic response elements as demonstrated by Exo-ChIP Seq (33). Moreover, mice with this impaired GR dimerization display induced resistance to the anti-inflammatory effects of both, exogenous and endogenous GCs in several inflammatory models (18–21, 34, 35).

Several clinical and experimental reports confirmed a critical role for endogenous GCs in maintaining bone mass (10, 12, 22, 36–38). The analysis of the basal bone phenotype in GR^dim^ mice used in the present study showed that, although cortical bone was unaffected, trabecular bone was enhanced in the femur but not the vertebrae. This is in part consistent with a previous study reporting no effect of impaired GR dimerization on trabecular bone mass of the vertebrae (22). Osteoblast and osteoclast counts did not differ in the femur between GR^dim^ and wild type mice, suggesting only a modest influence of the GR dimer during bone growth.

We observed in our investigation that thoracic trauma causes a systemic posttraumatic inflammation which confirmed previous studies (2, 9). IL-6 is considered as a hallmark cytokine of posttraumatic inflammation because multi-injured patients xhibit an increased IL-6 level within the first 24 h (39). Accordingly, although not statistically significant, IL-6 levels were increased in wild type mice upon additional thorax trauma. Studies using similar experimental settings in rats recorded increased IL-6 levels after 24 h (5, 9, 40). IL-6 was described to play a pathophysiological role in the impairment of fracture healing induced by severe trauma in mice (41, 42). In the present study, we observed either no or mild effects of thoracic trauma on IL-6 levels in plasma or BAL. This could possibly be explained by the different background strain used in the present study (43, 44). In contrast to our previous studies (41, 42), we here used BALB/c mice, because GR^dim^ mice in the C57BL/6 background do not survive. In the context of trauma-induced compromised fracture healing, plasma IL-6 levels in the GR^dim^ mice were not elevated. This contrasts with previous studies describing a failure to reduce IL-6 in GR^dim^ mice because they were resistant to the anti-inflammatory effects of not only endogenous GCs in sepsis models (21) but also of exogenous GCs treatment in the context of antigen-induced arthritis (34). This could be a characteristic of the combined trauma model resembling a milder inflammation than experimental septic shock models.

In the early fracture hematoma, MIP-1α was elevated in mice with impaired GR dimerization in the context of combined trauma compared to wild type mice. Similarly, fibroblast-like synoviocytes from GR^dim^ mice were resistant to dexamethasone-mediated MIP-1α suppression during experimental serum-induced arthritis (45). Reports on the role of MIP-1α on mesenchymal stem cell homing and migration to the injury site (46) but also on the enhancement of osteoclast differentiation and activation (47–49) suggest a beneficial role of MIP-1α on fracture healing outcome in our model. While systemic inflammation leads to hyper-inflammation in GR^dim^ mice (20, 21, 35), inflammation was not increased in the early fracture hematoma after combined injury. This observation correlates with models of contact allergy and arthritis, where despite the non-responsiveness of GR-deficient mice to exogenous GC treatment, these GR^dim^ mice were not hyper-responsive to inflammation as a response to endogenous GCs (18, 34). In conclusion, impaired GR dimerization affected inflammation in the circulation and marginally in the hematoma.

Flow cytometry analysis of the blood and fracture hematoma in GR^dim^ mice did not reveal major alterations in the immune cell composition 24 h post injury. This observation is partially in accordance with the cytokine measurements in the same compartments, because we did not observe major systemic inflammation.

In models of acute lung injury, GCs reduced inflammation *via* the induction of anti-inflammatory macrophages (50) and the presence of dimeric GR in macrophages was required to reduce vascular leakage of the endothelium (19). Our investigations indicated an upregulation of genes associated with a pro-inflammatory macrophage phenotype in the injured lungs of GR^dim^ mice (e.g., *Cd86*). Anti-inflammatory associated genes were clearly reduced in the combined treatment. *Il4* induction as an anti-inflammatory marker was less pronounced and *Chil3* was further reduced in GR^dim^ mice. Throughout the healing time, the effects on these markers were less prominent.

*Chil3* exhibits anti-inflammatory functions and participates in lung tissue repair (51–53). Likewise, in our model, *Chil3* gene expression was down regulated in mice with impaired GC signaling (GR^dim^) regardless of injury type, revealing a potential role of the GR dimer in *Chil3* regulation in the lung. This is in agreement with the concept that GCs induce rather an anti-inflammatory phenotype than just suppression of pro-inflammatory mediators (54, 55).

A study in human monocytes suggest that the main GC effects are not the suppression of pro-inflammatory mediators but rather the induction of an anti- inflammatory phenotype in monocytes and the promotion of their survival by prolonging the activation of the ERK/MAPK pathway (54). Moreover, transient presence of the macrophage chemokine MCP-1 was proven to be important for activation of anti-inflammatory macrophages and later on, to be beneficial for the lung contusion outcome (56). In line with these findings, we speculate that reduced expression of anti-inflammatory macrophage markers in GR^dim^ mice is linked to the reduced MCP-1 levels in the BAL.

Our investigations in the lungs provide insights for the function of the GR dimer in the inflammatory response towards thoracic trauma and strengthens its previously established role as mediator of GC-mediated anti-inflammatory effects. Current data do not allow conclusions about the lung repair outcome and further investigations are necessary to understand the effects of endogenous GCs during lung repair and its impact on fracture healing.

We have previously demonstrated a critical role of the GR during endochondral ossification in the context of isolated fracture healing (12). Interestingly, in the present study, impaired GR dimerization did not affect isolated fracture healing, indicating an important role of the GR monomer in mediating GC effects during endochondral ossification. By contrast, the GR dimer had a detrimental role during trauma-induced impaired fracture healing.

Regarding the outcome of impaired fracture healing after concomitant thoracic trauma, our model recapitulates the clinical (2) and experimental observations (5, 9). Indeed, and similarly to previous reports, biomechanical properties, cortical bridging score, and mineralization levels of the fracture callus at day 23 correlated with previous reports describing trauma-induced compromised fracture healing (27, 41, 57), whereas GR^dim^ mice displayed non-compromised healing. In contrast to GR^dim^ mice, wild type mice exhibited high cartilage content after combined injury. Intriguingly, there was no difference in osteoblast or osteoclast counts that could explain the different healing outcomes. Successful bone repair depends on effective cartilage-to-bone transition. Similar to a previous report (41), endochondral ossification in wild type mice was impaired upon combined injury because the callus bone content was significantly reduced 10 days later, whereas GR^dim^ mice did not display a reduced bone content.

Interference of the early inflammatory response with the healing cascade was described to be in part responsible for trauma-induced compromised fracture healing. Indeed, Recknagel et al. reported early alterations in neutrophil and monocyte infiltrations into the fracture callus and subsequent impairment of endochondral ossification (9). However, to what extent intact GR dimerization confers protection against thoracic trauma-impaired fracture healing *via* modulation of inflammation is unclear. Given the rather modest effects on the inflammatory response, this needs further investigation, possibly also in other murine backgrounds.

One possible cause could be that the high bone content observed in GR^dim^ mice upon combined trauma could also result from an accelerated bone-to-cartilage transformation. Accordingly, osteoblast numbers were higher in GR^dim^ mice compared to wild type mice 10 days post-combined trauma. This raises the possibility that GC signaling through the GR dimer would act on osteoblast progenitors and decrease their differentiation and/or proliferation in the context of trauma, thus leading to impaired endochondral ossification. In agreement with this, Rauch et al. reported an influence of dimeric GR on osteoblast proliferation rather than differentiation (22). Although intact GR dimerization was also described to be necessary for GC action on osteoclasts (58), we did not observe any changes in osteoclast numbers or surfaces throughout the investigated time in both genotypes.

A limitation of the study is that we do not clarify the exact molecular interactions and mechanisms that explain the adverse effects by the GR dimer. This requires further studies and may go beyond the regulation of inflammatory mediators or factors of bone cell differentiation. However, building on these data we and others are currently exploiting novel GR dimer gene regulation that is detrimental for trauma impact on fracture healing.

In conclusion, our data demonstrate a redundant role for intact GR dimerization during isolated fracture healing. However, in contrast to previous studies describing beneficial effects of the GR dimer in inflammatory models, we report here a negative effect of the intact GR dimerization in trauma-induced compromised fracture healing potentially by mediating the interaction of the thoracic trauma-induced systemic inflammation with the local inflammatory process at the fracture site. This study demonstrates a partial detrimental role of endogenous GCs/GR action on thoracic trauma-impaired fracture healing.

## Data Availability Statement

The original contributions presented in the study are included in the article/**Supplementary Material**, further inquiries can be directed to the corresponding author/s.

## Ethics Statement

The animal study was reviewed and approved by Regierungspräsidium Tübingen.

## Author Contributions

AI, JT, YH, and AR designed the study. AR, YH, SL, A-KD, BK, and KK conducted the experiments. AI, JT, YH, and AR analyzed and interpreted the data. YH drafted the manuscript. JT and AI revised the paper. All authors contributed to the article and approved the submitted version.

## Funding

This study was financed by the German Research Foundation in the context of the Collaborative Research Center 1149 – Project-ID 251293561 – “Danger Response, Disturbance Factors and Regenerative Potential after Acute Trauma” (CRC 1149, C02, INST 40/492-2).

## Conflict of Interest

The authors declare that the research was conducted in the absence of any commercial or financial relationships that could be construed as a potential conflict of interest.
