# Supplementary material for: Intact Glucocorticoid Receptor Dimerization Is Deleterious in Trauma-Induced Impaired Fracture Healing
Source: Front Immunol. 2021 Feb 17;11:628287. doi: 10.3389/fimmu.2020.628287 (PMC7927427; doi:10.3389/fimmu.2020.628287)
Supplement: Supplementary file 2 [file DataSheet_2.docx]

Supplementary Figures


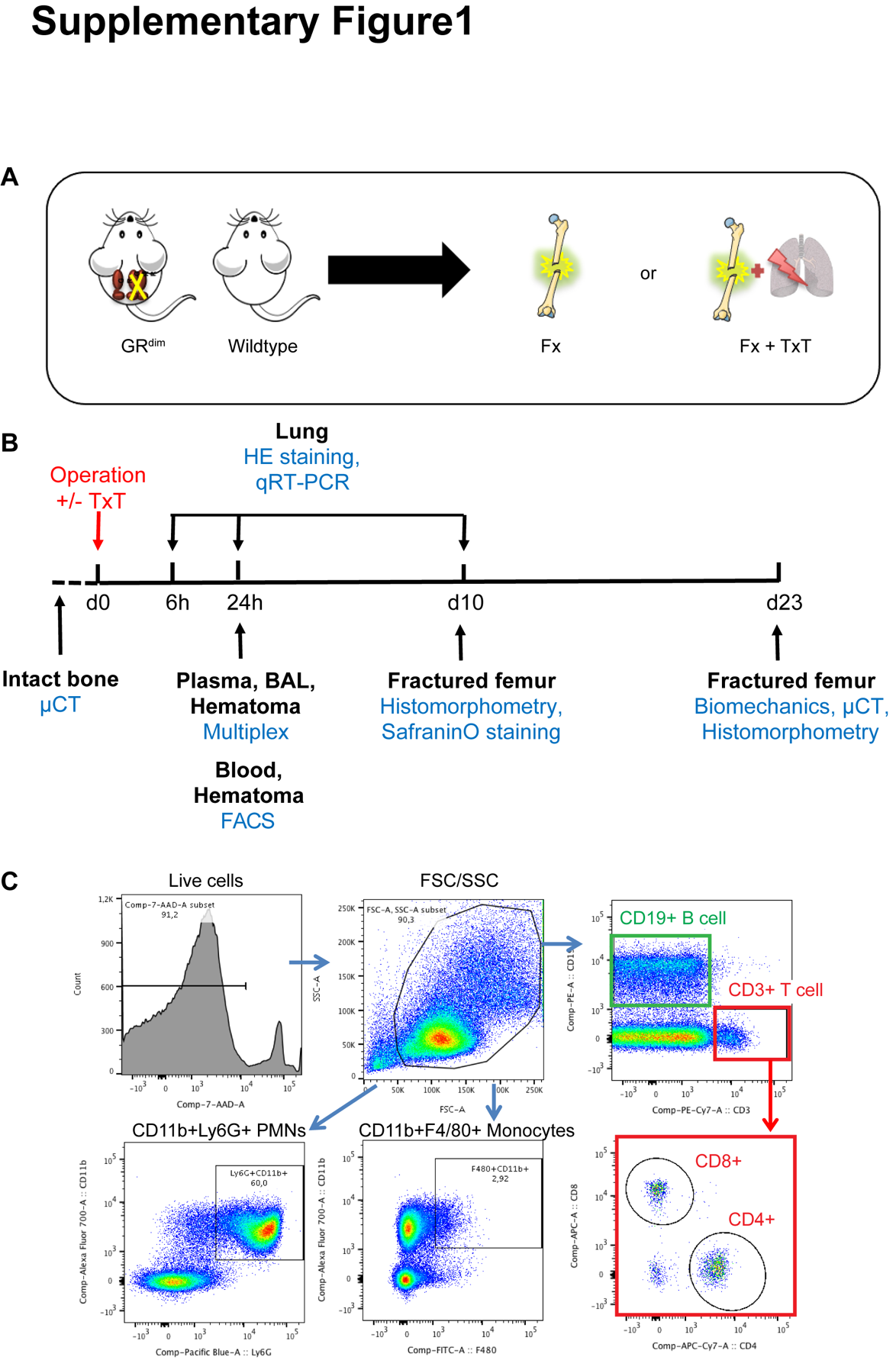


**Supplementary Figure 1.** **Study design and FACS gating strategy. (A, B)** Femur osteotomy with or without thoracic trauma was performed on 14-week old male GR^dim^ mice and littermate wildtype controls (A). Mice were euthanized at different time points during fracture healing and samples were analyzed as indicated (B). **(C)** FACS gating strategy for B cells (CD19^+^), T cells (CD3^+^), T-helper cells (CD3^+^CD4^+^), Cytotoxic T-cells (CD3^+^CD8^+^), PMNs (CD11b^+^Ly6G^+^) and Monocytes (CD11b^+^F4/80^+^).


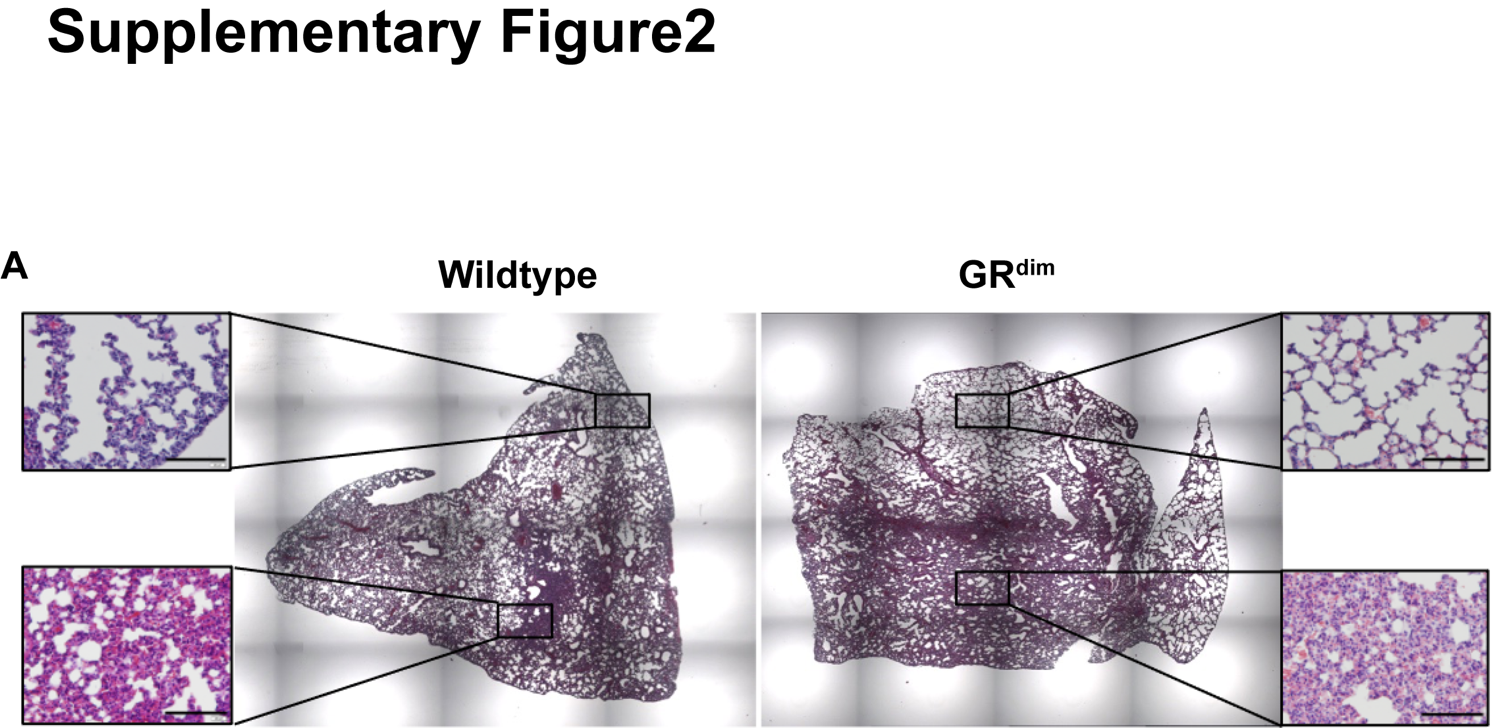


**Supplementary Figure 2.** **Thorax trauma affects lung structure and initiate a sustained inflammation in a model of thoracic trauma compromised fracture healing.** **(A)** H&E staining of lung sections illustrate the structural damage and the inflammation induced by the thoracic trauma 10 days later both in wild type and GR^dim^ mice. Lungs exhibit a patchwork injury pattern with both injured and less injured areas magnified. Scale bar in magnification micrographs 200 μm.
