# Supplementary material for: Intact Glucocorticoid Receptor Dimerization Is Deleterious in Trauma-Induced Impaired Fracture Healing
Source: Front Immunol. 2021 Feb 17;11:628287. doi: 10.3389/fimmu.2020.628287 (PMC7927427; doi:10.3389/fimmu.2020.628287)
Supplement: Supplementary file 3 [file Table_1.docx]

**Supplementary Table 1 qRT-PCR primer sequences target**

| Target gene | Forward 5' → 3' | Reverse 3' → 5' |
| --- | --- | --- |
| Gapdh | CCCATTCTCGGCCTTGACTGT | GTGGAGATTGTTGCCATCAACGA |
| *Il6* | AAACCGCTATGAAGTTCCTCTCTGC | AGCCTCCGACTTGTGAAGTGGT |
| *iNOS* | GAGACAGGGAAGTCTGAAGCAC | CCAGCAGTAGTTGCTCCTCTTC |
| *CD86* | TCAGTTACTGTGGCCCTCCT | GGCTCTCACTGCCTTCACTC |
| *Il4* | GGAGCCATATCCACGGATGCGAC | CATCTCCGTGCATGGCGTCCC |
| *Ym1* | CTGGGTCTCGAGGAAGCC | AGTGAGTAGCAGCCTTGGAA |
| *Il13* | CCAGGGCCGGTGCCAAGATC | AAGGGGCCGTGGCGAAACAG |
